# Lightweight Hotspot Detection Model Fusing SE and ECA Mechanisms

**DOI:** 10.3390/mi15101217

**Published:** 2024-09-30

**Authors:** Yanning Chen, Yanjiang Li, Bo Wu, Fang Liu, Yongfeng Deng, Xiaolong Jiang, Zebang Lin, Kun Ren, Dawei Gao

**Affiliations:** 1 Beijing Smartchip Microelectronics Technology Co., Ltd., Beijing 100192, China; chenyanning@sgchip.sgcc.com.cn (Y.C.); wubo@sgchip.sgcc.com.cn (B.W.); liufang@sgchip.sgcc.com.cn (F.L.); dengyongfeng@sgchip.sgcc.com.cn (Y.D.); 2 College of Integrated Circuits, Zhejiang University, Hangzhou 311200, China; 22341032@zju.edu.cn (Y.L.); 22141053@zju.edu.cn (X.J.); linzb@zju.edu.cn (Z.L.)

**Keywords:** hotspot detection, lithography, deep learning, convolutional neural network (CNN), lightweight model

## Abstract

In this paper, we propose a lightweight lithography machine learning-based hotspot detection model that integrates the Squeeze-and-Excitation (SE) attention mechanism and the Efficient Channel Attention (ECA) mechanism. These mechanisms can adaptively adjust channel weights, significantly enhancing the model’s ability to extract relevant features of hotspots and non-hotspots through cross-channel interaction without dimensionality reduction. Our model extracts feature vectors through seven convolutional layers and four pooling layers, followed by three fully connected layers that map to the output, thereby simplifying the CNN network structure. Experimental results on our collected layout dataset and the ICCAD 2012 layout dataset demonstrate that our model is more lightweight. By evaluating overall accuracy, recall, and runtime, the comprehensive performance of our model is shown to exceed that of ConvNeXt, Swin transformer, and ResNet 50.

## 1. Introduction

As the feature sizes of transistors continue to scale down, severe distortions occur in wafer patterns at wavelengths of 193 nm and below. This phenomenon, known as the Optical Proximity Effect (OPE), describes the deviation between the mask pattern and the wafer surface pattern transfer. Despite the application of various resolution enhancement techniques (RETs) [1], such as Optical Proximity Correction (OPC) [2], Phase-Shift Masking (PSM) [3], and Sub-Resolution Assist Features (SRAFs) [4], manufacturing defects [5] still occur under the influence of OPE. These regions, where defects are likely to form, are referred to as lithography hotspots. Therefore, detecting lithography hotspots before transferring the mask pattern to the wafer is crucial.

Detecting hotspots before lithography can significantly reduce defects [6]. Currently, there are three main methods for hotspot detection: lithography simulation [7,8], pattern matching, and machine learning. Lithography simulation accurately simulates the lithography process but incurs high computational costs. Given that actual hotspot areas constitute only a small part of the entire chip, existing methods typically perform a quick classification to extract candidate hotspots for lithography simulation.

Pattern matching involves establishing a set of hotspot layout patterns to identify shapes in new layouts that match these patterns as hotspots [9,10,11,12,13]. Wen et al. [11] proposed a fuzzy matching model that combines pattern matching and machine learning techniques to dynamically adjust the fuzzy areas around known hotspots. Although pattern matching addresses runtime issues, it cannot accurately detect unknown hotspot patterns.

Machine learning-based techniques have proven effective in various applications in IC manufacturing, including mask optimization, hotspot detection, and lithography validation [14,15,16]. These techniques maintain high prediction accuracy [17] through effective layout sampling methods. Typically, models are trained on features extracted from a batch of labeled data and then used to predict hotspots in new layout patterns.

As masks for VLSI become increasingly complex, traditional machine learning techniques struggle to accurately model the vast amounts of layout data. Advanced layout feature extraction methods, such as density [18] and Class Center Similarity (CCS) [19], inevitably suffer from spatial information loss. To overcome these challenges, deep convolutional neural networks (CNNs), known for their strong image recognition capabilities, have been applied in hotspot detection [20,21,22]. For instance, Yang et al. [23] studied a deep CNN that addresses data imbalance issues and achieves high classification accuracy. To address the problem of limited labeled data, Chen et al. [24] developed a semi-supervised neural network. Additionally, Jiang et al. [25] proposed a binarized neural network to further enhance the performance of the detector.

Deep learning-based methods for hotspot detection can significantly improve both detection accuracy and efficiency, accurately identifying hotspots and reducing wafer defects. However, the limited widespread application of deep learning methods in chip manufacturing can be attributed to several factors:

(1) Detection Accuracy for Large-Scale Layouts: Current deep learning models require further improvement in detection accuracy when applied to large-scale layouts.
(2) Resource Utilization: Existing models often have large parameter sizes and complex network architectures, leading to excessive resource utilization in practical deployment, necessitating further efficiency enhancements.

In this paper, we designed a lightweight network structure based on a CNN and incorporated the SE attention mechanism and the ECA mechanism to develop a lightweight lithographic hotspot detection (LHD) model. This model reduces complexity and computational costs. We employed global average pooling to aggregate the spatial feature information of hotspot images, thereby enhancing the network’s stability. Experimental results on the collected dataset demonstrate that the LHD model achieves high accuracy and speed in hotspot detection across various types of hotspots, addressing the main drawbacks of deep learning-based hotspot detection. Furthermore, experimental results for the ICCAD 2012 layout dataset indicate that the LHD model outperforms widely used classical network structures, including ConvNeXt [26], Swin Transformer [27], and ResNet50 [28], in hotspot detection performance, in terms of accuracy and runtime.

## 2. Preliminary

ConvNeXt, Swin Transformer, and ResNet50, based on convolutional neural networks, have demonstrated excellent performance in classification competitions and have been widely adopted for various image classification tasks. Consequently, these models have also achieved notable results in hotspot detection. However, due to their numerous convolutional layers, extensive convolutions, and complex deep neural network architectures, they possess a large number of parameters and high computational complexity. To mitigate this, dimensionality reduction of feature vectors is often employed during feature extraction. Nonetheless, this reduction can compromise feature extraction capabilities, leading to decreased classification accuracy. Therefore, designing a classification model that maintains high accuracy while significantly reducing computational complexity is of paramount importance.

In deep learning classification models, standard evaluation metrics include accuracy, recall, precision, F1 score, and runtime. These metrics are utilized to assess the performance of an LHD model in hotspot detection and conduct comparative experiments with traditional classification models. Hotspot detection results can be categorized into the following scenarios:

True Positive (TP): Correctly identifying hotspot graphics as hotspots.

True Negative (TN): Correctly identifying non-hotspot graphics as non-hotspot.

False Positive (FP): Incorrectly identifying non-hotspot graphics as hotspots.

False Negative (FN): Incorrectly identifying hotspots as non-hotspot graphics.

The definitions of accuracy, recall, precision, and F1 score are as follows:

$$accuracy=\frac{TP+TN}{TP+TN+FP+FN}$$

$$recall=\frac{TP}{TP+TN}$$

$$precision=\frac{TP}{TP+FP}$$

$$F1=2*\frac{\left(precision*recall\right)}{precision+recall}$$

Among these metrics, accuracy represents the proportion of correctly predicted hotspot and non-hotspot regions out of the total samples. Recall represents the proportion of hotspot regions correctly predicted by the model out of the actual hotspot regions. Precision represents the proportion of correctly predicted hotspot regions out of the total hotspot regions predicted by the model. The F1 score is the harmonic mean of recall and precision, providing a balanced metric of overall performance. Runtime indicates the detection duration consumed per unit chip layout area. A lower runtime signifies less detection time per unit area, higher model detection efficiency, and lower time and economic costs. All of these evaluation metrics range between 0 and 1, with values closer to 1 indicating better model performance.

The Softmax function outputs a probability distribution over class predictions. After applying the Softmax operation, it becomes easier to compute the error with respect to discrete labels. In fact, the class distribution can also represent true labels: for a given sample i, a vector *P*(*x^i^*) ∈ *R^q^* is generated, where the *P*(*x_i_*) element (corresponding to the discrete class of sample i) is set to 1 and all other elements are set to 0. This allows the training objective to make the predicted probability distribution *Q*(*x_i_*) as close as possible to the true label distribution *P*(*x_i_*).

When using mean squared error (MSE) as the loss function, the prediction error is minimized to a very low level. However, such low error control is not necessary to achieve correct classification. Therefore, we adopt cross-entropy, a loss function that is better suited for measuring the difference between two probability distributions.

$$H\left(P,Q\right)=-\sum_{i=1}^{n}P\left(x_{i}\right)\log\left(Q\left(x_{i}\right)\right)$$

For the same random variable xxx, there are two independent probability distributions, *P* and *Q*, where *P* represents the true probability distribution and *Q* represents the predicted probability distribution.

Cross-entropy is commonly used in classification tasks alongside the Softmax function. Softmax outputs a predicted probability distribution, which is then fed into the cross-entropy function, allowing the model to adjust based on the cross-entropy value. In binary classification, the cross-entropy loss function takes the following form:

$${BCE}_{Loss}=-(ylog\left(Q\left(x\right)\right)+\left(1-y\right)log(1-Q(x)))$$

where *y* represents the true label (either 0 or 1), as mentioned for *P*(*x_i_*) above, and Q(x) denotes the predicted probability for the sample. We adopt this loss function as the loss function for the LHD model.

## 3. LHD Model Fusing SE and ECA Mechanisms

### 3.1. SE Model and ECA Model

Each output value of the convolution operation depends solely on adjacent pixel data in the input feature map, integrating spatial and channel information via local receptive fields. However, this approach often leads to redundancy and insufficient extraction of channel features in deep neural networks. To reduce this issue, Hu et al. [29] introduced the “SE module”, which adaptively adjusts the weights of each channel in the feature map based on direct inter-channel dependencies, with minimal additional computational cost. This adaptive feature recalibration mechanism effectively addresses the limitations of traditional deep neural networks in channel feature extraction, thereby enhancing overall network performance.

**Figure 1:**
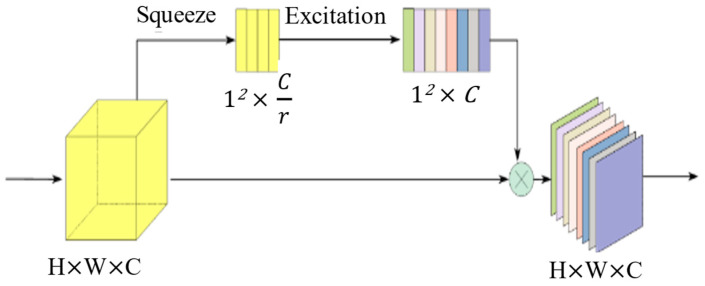
Schematic diagram of the SE module.

In this study, SE modules are incorporated between convolutional and pooling layers to adaptively extract image features from the encoder convolution’s feature mapping [30]. By leveraging the channel attention mechanism, the network can effectively prioritize features relevant to specific classification tasks. Figure 1 illustrates the fundamental structure of an SE module: the convolutional layer extracts three-dimensional feature maps from each channel (with dimensions H, W, and C), which are then subjected to global average pooling, and a fully connected layer is employed to compress the channel dimensions of the feature map, a process referred to as “Squeeze”. Following this, the “Excitation” phase is conducted, wherein a nonlinear transformation is applied to obtain the weights for each feature channel. These weights are then used to re-calibrate the output feature map. The parameter r represents the reduction ratio, which encodes the interdependencies between the channels [31].

The SE attention mechanism offers several advantages compared to other attention mechanisms. The SE module extracts global spatial information via global average pooling (Squeeze) and generates channel weights through fully connected layers (Excitation), assigning different attention weights to different channels. This makes it easy to integrate into existing Convolutional Neural Networks (CNNs). The SE module has low computational overheads, as it primarily involves channel-level reweighting through a series of fully connected layers, without requiring complex matrix multiplications or multihead operations. The SE mechanism enhances the representation of important features and suppresses irrelevant ones by adaptively adjusting channel weights, leading to significant performance improvements. Moreover, the SE module focuses on channel attention, addressing CNNs’ limitations in capturing inter-channel relationships. By leveraging channel attention for feature extraction, the SE module helps improve the model’s generalization ability across various datasets and tasks.

To enhance performance without increasing model complexity, Wang et al. [32] proposed the ECA module. Unlike dimensionality reduction techniques, the ECA module improves the model’s feature representation capability through effective cross-channel interactions. This approach significantly boosts model performance while maintaining higher computational efficiency, fewer parameters, and a lightweight structure.

The ECA module necessitates delineating the interaction coverage range to capture local cross-channel interactions efficiently. However, manually adjusting the optimal interaction coverage range consumes significant computational resources. Enhancing high-dimensional (low-dimensional) channels within the CNN structure facilitates the sharing of convolutions over long distances (short distances) for a given number of groups. The coverage range of channel interactions (represented by the size of the 1D convolution kernel *k*) is directly proportional to the channel dimension C, where a mapping function φ exists between *k* and *C*.

$$C=\varphi \left(k\right)$$

**Figure 2:**
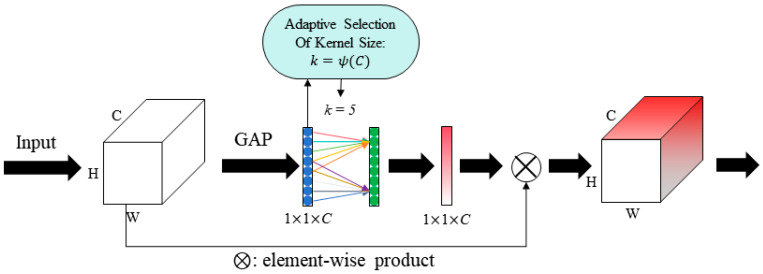
Schematic diagram of the ECA module.

Figure 2 illustrates that following global average pooling within each channel (while preserving feature map dimensionality), the ECA module integrates information from each channel and its adjacent *k* channels. A larger *k* widens the scope of inter-channel information interaction. In practical implementations, ECA can be effectively realized using a one-dimensional convolution of size *k*, thereby greatly enhancing the model’s capability to extract crucial features from the layout, with minimal computational overheads.

### 3.2. LHD Model Structure

**Figure 3:**
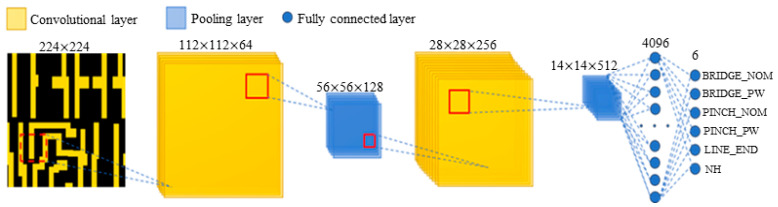
CNN extracts layout image features.

Figure 3 illustrates that CNNs extract features from images and predict hotspot regions through a sequence of convolutional layers, pooling layers, and fully connected layers. Convolutional layers employ learnable kernels to locally extract features from input images, capturing spatial relationships among hotspot regions. Pooling layers subsequently reduce feature map dimensionality while preserving key feature information. Finally, fully connected layers map these extracted features to the output layer, where the Softmax activation function generates probability predictions for hotspots.

Model training relies on a labeled lithography image dataset, where the model computes errors between predicted and true labels, optimizing this through a defined loss function. Post-training, the model predicts hotspot regions in new lithography images, with outputs threshold to identify hotspot regions. Leveraging the characteristics of CNNs, the deep learning model achieves hotspot detection through effective feature extraction and prediction. Trained on extensive datasets, the model demonstrates efficient and accurate hotspot detection capabilities in lithography images.

**Figure 4:**
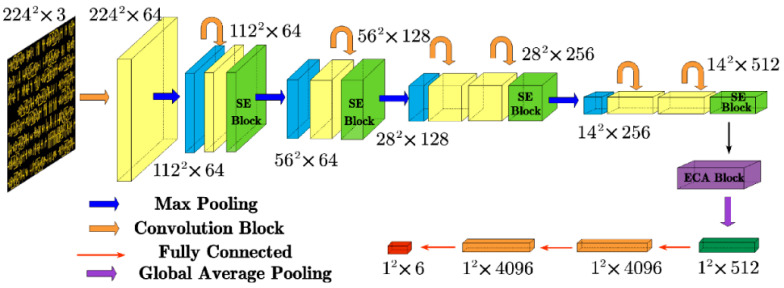
Structural design of the LHD model.

The network structure of the LHD model is shown in Figure 4. To achieve a lightweight model implementation without compromising detection accuracy, the LHD model integrates SE and ECA modules. These enhancements are accompanied by a reduction in redundant convolutional and fully connected layers during network construction. The incorporation of these modules allows the network to prioritize relevant features in hotspot and non-hotspot graphics, thereby minimizing attention to redundant background information. This streamlined approach effectively lowers network complexity while simultaneously enhancing accuracy.

The design principles of the LHD model encompass three key aspects:

(1) Lightweight Convolutional Neural Network Structure: The model is built upon fundamental convolutional network elements, featuring seven CNN convolutional layers and four pooling layers for extracting feature map vectors. This structure is streamlined with three fully connected layers mapping to the output, ensuring efficient connectivity.
(2) Integration of the SE Attention Mechanism: By incorporating the SE attention mechanism, the model adjusts the weights of each channel within feature maps derived from convolution operations. This integration allows the model to autonomously discern the significance of different channels, effectively capturing essential information and thereby reducing computational complexity.
(3) Integration of ECA Modules: The inclusion of ECA modules enhances cross-channel interactions without altering feature map dimensions. This fosters close dependencies among channels, achieving a balanced trade-off between model performance and complexity.

### 3.3. Data Preparation

**Figure 5:**
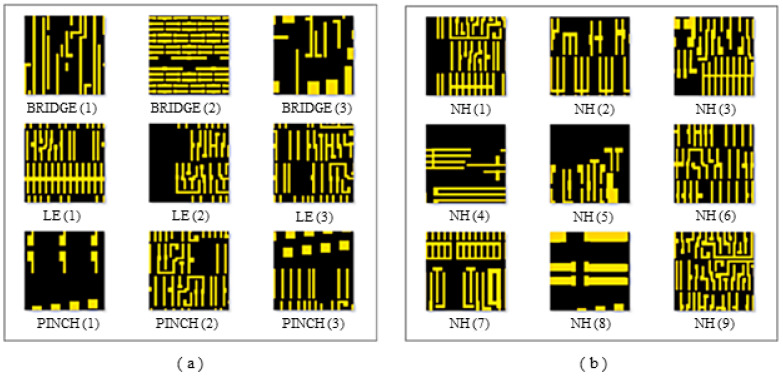
Collected layout dataset: (**a**) hotspot layout image; (**b**) non-hotspot layout image.

Figure 5 depicts the collected dataset utilized for training and evaluation in this study. The dataset consists of 16,251 polysilicon gate-layer layouts sourced from a wafer fabrication facility. These layouts specifically pertain to 55 nm polysilicon gate-layer mask layouts, covering an area of 673,446 μm^2^. The collected data are categorized into six classes: BRIDGE_NOM (standard condition bridge nominal), BRIDGE_PW (process variation bridge nominal), PINCH_NOM (standard condition pinch nominal), PINCH_PW (process variation pinch nominal), LINE END (line-end shortening error), and NH (non-hotspot) classes; the counts are 283, 269, 736, 697, 796, and 13,470 respectively. The training-to-testing set ratio is 8:2. SEM (Scanning Electron Microscope) images obtained post-lithography are matched with corresponding mask layout images, classified based on the discrepancy between measured CD values and target values to construct the dataset.

### 3.4. Experimental Methodology

**Figure 6:**
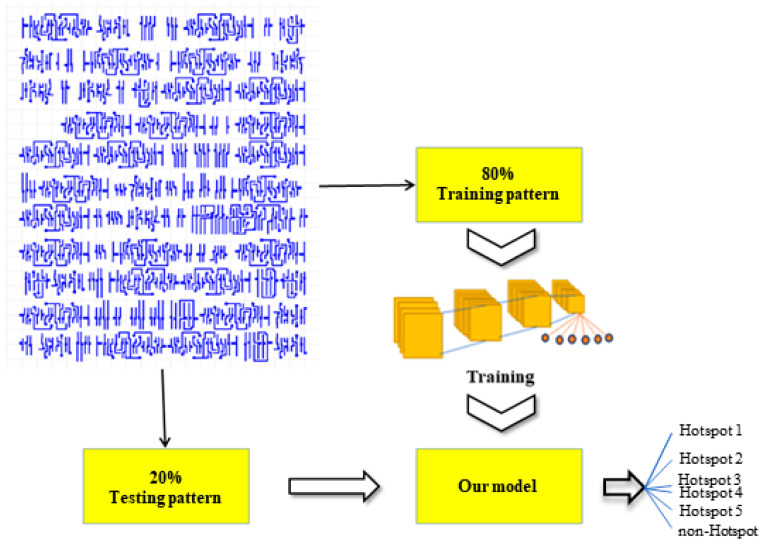
Experiment methodology for classic models and LHD model.

In this study, a supervised deep learning approach using PyTorch 1.13.1 was employed for hotspot detection. Figure 6 illustrates the experiment methodology, encompassing training and testing phases, the main methodology is outlined as follows:

(1) Data Preparation: Mask patterns were cropped and converted into images to construct the dataset. The data were labeled into five categories of hotspots and one category of non-hotspots based on simulation outcomes.
(2) Training and Testing Split: Eighty percent of the dataset was allocated for training the model. The remaining 20% was reserved for testing and evaluating the model’s predictive performance on hotspot types.
(3) Algorithm Optimization and Model Enhancement: Techniques for algorithm optimization and model refinement were implemented to improve hotspot detection accuracy. Existing neural network architectures were assessed and compared to determine their efficacy in this specific task.

This structured approach ensures robust training, thorough evaluation, and iterative improvement of the deep learning model for effective hotspot detection in semiconductor manufacturing processes.

When lithography hotspot images are fed into a CNN, the network extracts image features through convolutional kernels and integrates them to produce an output [33]. Various layout patterns serve as input data to train the network. As a supervised learning approach, all graphical data used for training the model are labeled. Feature extraction is automated through backpropagation and optimization techniques.

During hotspot detection, the trained model analyzes layout graphics to identify potential hotspots. By applying a predefined threshold to the model’s output, we determine the presence of hotspots in the image. A CNN is utilized for classifying graphical data into five distinct types of hotspot and non-hotspot categories. This methodology ensures systematic detection and classification of lithography hotspots, which is crucial for optimizing semiconductor manufacturing processes.

## 4. Results

We implemented our experiment in the Python programming language. An NVIDIA GeForce RTX 4090 GPU (NVIDIA Corporation, Clara, CA, USA) was utilized, running on the Windows system. The network model was coded in Python 3.8, with PyTorch serving as the deep learning framework. The Adam optimizer was employed for 200 iterations. The initial parameter settings included a learning rate of 0.00001, a weight decay of 0.05, and a dropout rate of 0.5.

**Figure 7:**
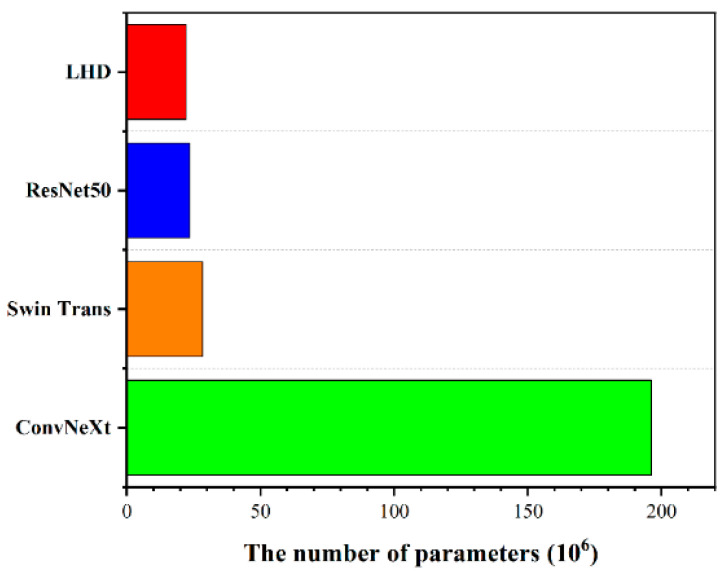
The parameter counts of four models.

Deep learning detection methods require significant computational resources and costs, so designing lightweight models has become a key consideration. Figure 7 shows the parameter count of four models, ranked from highest to lowest, as follows: ConvNeXt with 196,155,270 parameters, Swin Transformer with 28,265,032 parameters, ResNet50 with 23,520,326 parameters, and LHD with 22,132,548 parameters. In terms of model lightweightness, our LHD model is the most compact.

**Figure 8:**
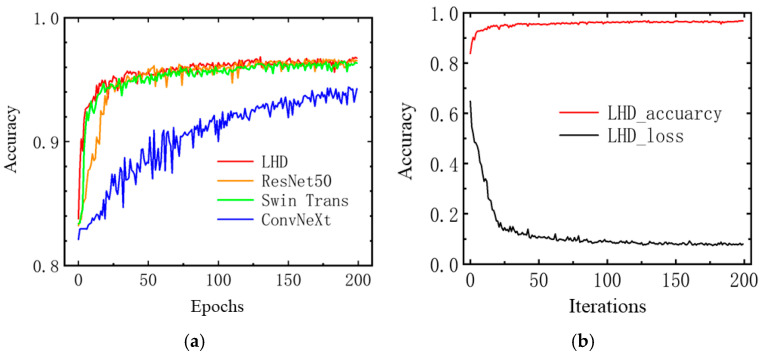
Accuracy trends with iterations on collected dataset. (**a**) Accuracy trends for Different Models; (**b**) Loss Function and Accuracy for the LHD Model.

Figure 8a demonstrates the convergence speed in terms of accuracy for four models on the training set of the collected dataset. It is evident that the Swin and LHD models converge more quickly, while the LHD and ResNet models achieve higher accuracy after convergence. Figure 8b provides a detailed view of the changes in the loss function and accuracy for the LHD model, showing that the loss function approaches convergence after 50 iterations, with a final value of 0.074. This indicates that the LHD model’s loss function decreases rapidly and converges during training.

**Figure 9:**
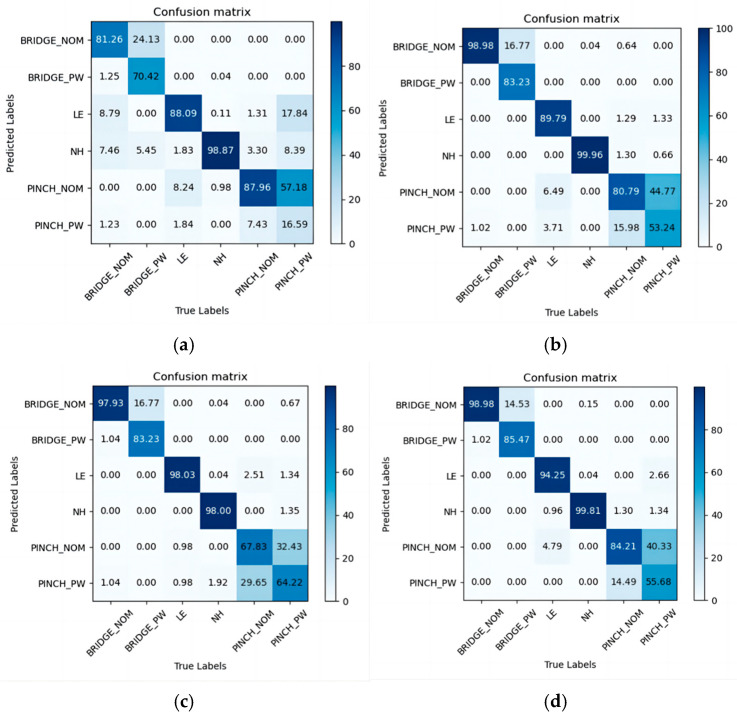
The accuracy confusion matrix of each model on the collected dataset: (**a**) ConvNeXt; (**b**) Swin Transformer; (**c**) ResNet50; (**d**) LHD.

We ran four models on the test dataset to obtain the prediction accuracy for each type of hotspot. Figure 9 shows the confusion matrices of the accuracy for these models on the collected dataset, with the diagonal elements representing the probability of accurate prediction for each class. The values on the diagonal in the figure represent the model’s accuracy for classifying each type of hotspot. The accuracy of the LHD model is better than that of ConvNeXt and Swin Transformer and nearly consistent with that of ResNet50. Table 1 displays the overall detection performance of the four models on the collected dataset. The LHD model achieved an Accuracy of 96.83%, outperforming ResNet50 (96.64%), Swin Transformer (96.49%), and ConvNeXt (94.39%). These results corroborate the findings from the confusion matrices mentioned earlier. During the detection phase, the LHD model required 4.95 h per square millimeter, which is 1.15 h per square millimeter less than that of the ResNet50 model. Although the LHD model exhibited slightly lower precision compared to ResNet50 by 0.85%, the combination of high detection accuracy and reduced cost makes the LHD model more suitable for practical hotspot detection tasks in lithography. Despite challenges posed by high similarity in the PINCH_NOM category, the model achieved an accuracy of 79.9%, surpassing the precision of the other models in this category.

To assess the generalization ability of the LHD model, we conducted experiments using the ICCAD 2012 mask layout dataset [34]. The specific distribution of the training and test sets in ICCAD 2012 is shown in Table 2. The ICCAD 2012 layout dataset is divided into two parts: a training dataset and a test dataset. Benchmark 1 corresponds to a 32 nm layout, whereas the other four benchmarks are 28 nm layouts. The layout areas for Benchmarks 1 through 5 are 12,516 μm^2^, 106,954 μm^2^, 122,565 μm^2^, 82,010 μm^2^, and 49,583 μm^2^, respectively. To address the issue of data imbalance mentioned in [34], we applied data augmentation techniques during the training phase by flipping and mirroring the hotspot dataset. This approach helps to mitigate the imbalance problem.

Table 3 summarizes the results of these experiments. Across Benchmarks 1–4, the LHD model consistently exhibited higher accuracy compared to the other classic models. In Benchmark 5, characterized by a lower proportion of hotspots, all models demonstrated comparable detection accuracies and times. Notably, the LHD model showed superior mean precision and recall relative to the other classic models, while maintaining relatively lower detection times. Ref. [23] utilizes a CNN-based deep learning approach to automatically extract layout features and addresses the class imbalance between hotspot and non-hotspot samples through data augmentation techniques. Ref. [24] employs a semi-supervised self-paced multitask learning approach that leverages both labeled and unlabeled data. The method utilizes a self-paced multitask learning framework to enhance the model’s generalization capability. Compared to [23,24], our experimental results show superior accuracy, particularly in Benchmark 4 and Benchmark 5. These datasets contain fewer samples, highlighting the strength of our model in feature extraction. Additionally, our model’s accuracy consistently surpasses that of [24], demonstrating its generalization ability across various benchmarks.

Specifically, in Benchmark 3, the LHD model demonstrated significant enhancements in accuracy (2.34%), precision (7.97%), and recall (2.63%) compared to the ResNet50 model, along with a reduction in detection time by 1.81 $h∕mm^{2}$. These findings underscore the robust performance of the LHD model across diverse benchmarks.

## 5. Conclusions

We propose a lightweight LHD lithography hotspot detection method. Our LHD model integrates the SE attention mechanism and the ECA mechanism into a simplified convolutional neural network architecture. This lightweight structure significantly reduces the number of parameters and computational complexity, thereby lowering computational costs. The SE module enables adaptive adjustment of feature map channel weights, enhancing the network’s feature learning capability. Additionally, the ECA module improves feature extraction efficiency with minimal additional parameters, overcoming dimensionality reduction challenges observed in the SE mechanism. We evaluated the LHD model on common hotspot types, such as BRIDGE_NOM, BRIDGE_PW, PINCH_NOM, PINCH_PW, and LE. Our experiments demonstrated that the LHD model achieves superior runtime efficiency and accuracy compared to three classic models: ResNet50, Swin Transformer, and ConvNeXt. To assess the generalization ability of our approach, we tested the LHD model using the ICCAD 2012 layout dataset, where it consistently outperformed the aforementioned classic models. These results highlight that our lightweight LHD lithography hotspot detection method not only surpasses traditional models in performance but also maintains efficiency in its network architecture.

## Figures and Tables

**Table 1:** Comparison of the performances on the collected dataset.

Model	Accuracy (%)	Precision (%)	Recall (%)	Runtime (h/mm^2^)
ConvNeXt	94.39	77.74	93.56	7.67
Swin Trans	96.49	95.00	96.33	7.75
ResNet50	96.64	94.94	94.91	6.10
**Our LHD**	**96.83**	**94.09**	**96.69**	**4.95**

**Table 2:** ICCAD 2012 benchmark statistics.

Name	Training Data			Testing Data		
	Hotspot	Non-Hotspot	Total	Hotspot	Non-Hotspot	Total
Benchmark 1	99	340	439	226	4679	4905
Benchmark 2	174	5285	5459	498	41,298	41,796
Benchmark 3	909	4643	5552	1808	46,333	48,141
Benchmark 4	95	4452	4547	177	31,890	32,067
Benchmark 5	26	2176	2202	41	19,327	19,368
Total	1303	16,896	18,199	2750	143,527	146,277

**Table 3:** Comparison of detection results for each model on the ICCAD 2012 dataset.

Pattern	Model	Accuracy (%)	Precision (%)	Recall (%)	Runtime (h/mm^2^)
Benchmark 1	ConvNeXt	81.61	66.23	78.89	10.89
	Swin Trans	89.16	90.16	95.11	10.72
	ResNet50	90.80	93.86	93.34	10.67
	[23]	100.0	-	-	1.11
	[24]	-	-	-	-
	**Our LHD**	**95.40**	**97.57**	**97.13**	**10.65**
Benchmark 2	ConvNeXt	96.79	53.43	96.72	25.84
	Swin Trans	97.43	69.95	97.69	26.74
	ResNet50	97.25	71.47	97.53	23.57
	**[23]**	**98.70**	**-**	**-**	**1.31**
	**[24]**	**97.99**	**-**	**-**	**-**
	**Our LHD**	**98.90**	**89.34**	**99.33**	**24.41**
Benchmark 3	ConvNeXt	91.52	71.99	90.20	26.74
	Swin Trans	93.51	75.69	92.18	26.74
	ResNet50	93.96	78.17	92.70	23.57
	[23]	98.0	-	-	1.24
	[24]	98.25	-	-	-
	**Our LHD**	**96.30**	**86.14**	**95.33**	**21.76**
Benchmark 4	ConvNeXt	97.91	48.96	97.91	36.58
	Swin Trans	98.46	87.54	98.96	37.26
	ResNet50	98.68	85.50	99.24	33.87
	[23]	94.50	-	-	1.17
	[24]	89.60	-	-	-
	**Our LHD**	**99.23**	**92.65**	**99.68**	**31.16**
Benchmark 5	ConvNeXt	99.09	49.41	98.33	54.90
	Swin Trans	99.09	49.41	98.33	54.90
	ResNet50	98.54	49.41	98.33	50.42
	[23]	95.10	-	-	1.48
	[24]	95.12	-	-	-
	**Our LHD**	**99.09**	**95.61**	**99.98**	**48.18**

## Data Availability

Data underlying the results presented in this paper are not publicly available at this time but may be obtained from the authors upon reasonable request.

## References

[1] Cecil T., Peng D., Abrams D., Osher S.J., Yablonovitch E. (2022). Advances in inverse lithography. ACS Photonics.

[2] Banerjee S., Agarwal K.B., Orshansky M. Simultaneous OPC and Decomposition for Double Exposure Lithography. Proceedings of the Optical Microlithography.

[3] Mack C.A. (2006). Field Guide to Optical Lithography.

[4] Viswanathan R., Azpiroz J.T., Selvam P. Process optimization through model based SRAF printing prediction. Proceedings of the Optical Microlithography.

[5] Yang H., Su J., Zou Y., Yu B., Young E.F. (2019). Layout Hotspot Detection With Feature Tensor Generation and Deep Biased Learning. IEEE Trans. Comput. Aided Des. Integr. Circuits Syst..

[6] Yu B., Gao J.R., Ding D., Zeng X., Pan D.Z. (2015). Accurate lithography hotspot detection based on principal component analysis-support vector machine classifier with hierarchical data clustering. J. Micro/Nanolithography MEMS MOEMS JM3.

[7] Kim J., Fan M. Hotspot detection on post-OPC layout using full-chip simulation-based verification tool: A case study with aerial image simulation. Proceedings of the 23rd Annual BACUS Symposium on Photomask Technology.

[8] Kahng A.B., Park C.H., Xu X. Fast Dual Graph Based Hotspot Detection. Proceedings of the Photomask Technology.

[9] Yao H., Sinha S., Chiang C., Hong X., Cai Y. Efficient Process-Hotspot Detection Using Range Pattern Matching. Proceedings of the 2006 IEEE/ACM International Conference on Computer-Aided Design.

[10] Yu Y.T., Chan Y.C., Sinha S., Jiang IH R., Chiang C. Accurate process-hotspot detection using critical design rule extraction. Proceedings of the 49th Annual Design Automation Conference.

[11] Wen W.Y., Li J.C., Lin S.Y., Chen J.Y., Chang S.C. (2014). A Fuzzy-Matching Model With Grid Reduction for Lithography Hotspot Detection. IEEE Trans. Comput. Aided Des. Integr. Circuits Syst..

[12] Tseng S.S., Chang W.C., Jiang I.H.-R., Zhu J., Shiely J.P. Efficient Search of Layout Hotspot Patterns for Matching SEM Images using Multilevel Pixelation. Proceedings of the Optical Microlithography XXXII.

[13] Roseboom E., Rossman M., Chang F.-C., Hurat P. Automated full-chip hotspot detection and removal flow for interconnect layers of cell-based designs. Proceedings of the Design for Manufacturability through Design-Process Integration.

[14] Jia N., Lam E.Y. Stochastic gradient descent for robust inverse photomask synthesis in optical lithography. Proceedings of the 2010 IEEE International Conference on Image Processing.

[15] Duo D., Bei Y., Ghosh J., Pan D.Z. EPIC: Efficient prediction of IC manufacturing hotspots with a unified meta-classification formulation. Proceedings of the 17th Asia and South Pacific Design Automation Conference.

[16] Xiao Z., Du Y., Tian H., Wong M.D., Yi H., Wong HS P., Zhang H. Directed Self-Assembly (DSA) Template Pattern Verification. Proceedings of the 51st ACM/EDAC/IEEE Design Automation Conference (DAC).

[17] Shim S., Chung W., Shin Y. Synthesis of Lithography Test Patterns through Topology-Oriented Pattern Extraction and Classification. Proceedings of the Design-Process-Technology Co-optimization for Manufacturability VIII.

[18] Matsunawa T., Gao J.R., Yu B., Pan D.Z. A New Lithography Hotspot Detection Framework Based on AdaBoost Classifier and Simplified Feature Extraction. Proceedings of the Design-Process-Technology Co-optimization for Manufacturability.

[19] Matsunawa T., Yu B., Pan D.Z. Optical Proximity Correction with Hierarchical Bayes Model. Proceedings of the Optical Microlithography.

[20] Krizhevsky A., Sutskever I., Hinton G.E. ImageNet Classification with Deep Convolutional Neural Networks. Proceedings of the 25th International Conference on Neural Information Processing Systems.

[21] Simonyan K., Zisserman A. (2014). Very Deep Convolutional Networks for Large-Scale Image Recognition. arXiv.

[22] Zeiler M.D., Fergus R. Visualizing and Understanding Convolutional Networks. Proceedings of the 13th European Conference.

[23] Yang H., Luo L., Su J., Lin C., Yu B. Imbalance Aware Lithography Hotspot Detection: A Deep Learning Approach. Proceedings of the Design-Process-Technology Co-Optimization for Manufacturability.

[24] Chen Y., Lin Y., Gai T., Su Y., Wei Y., Pan D.Z. (2020). Semisupervised Hotspot Detection with Self-Paced Multitask Learning. IEEE Trans. Comput. Aided Des. Integr. Circuits Syst..

[25] Jiang Y., Yang F., Zhu H., Yu B., Zhou D., Zeng X. Efficient Layout Hotspot Detection via Binarized Residual Neural Network. Proceedings of the 56th ACM/IEEE Design Automation Conference (DAC).

[26] Li H., Wang N., Yang X., Wang X., Gao X. A ConvNet for the 2020s. Proceedings of the IEEE/CVF Conference on Computer Vision and Pattern Recognition.

[27] Liu Z., Lin Y., Cao Y., Hu H., Wei Y., Zhang Z., Lin S., Guo B. Swin Transformer: Hierarchical Vision Transformer using Shifted Windows. Proceedings of the IEEE/CVF International Conference on Computer Vision (ICCV).

[28] Mukti I.Z., Biswas D. Transfer Learning Based Plant Diseases Detection Using ResNet50. Proceedings of the 4th International Conference on Electrical Information and Communication Technology (EICT).

[29] Hu J., Shen L. Squeeze-and-Excitation Networks. Proceedings of the 2018 IEEE/CVF Conference on Computer Vision and Pattern Recognition.

[30] Wang J., Lv P., Wang H., Shi C. (2021). SAR-U-Net: Squeeze-and-excitation block and atrous spatial pyramid pooling based residual U-Net for automatic liver segmentation in Computed Tomography. Comput. Methods Programs Biomed..

[31] Roy A.G., Navab N. (2019). Recalibrating Fully Convolutional Networks with Spatial and Channel “Squeeze and Excitation” Blocks. IEEE Trans. Med. Imaging.

[32] Wang Q., Wu B., Zhu P., Li P., Zuo W., Hu Q. ECA-Net: Efficient Channel Attention for Deep Convolutional Neural Networks. Proceedings of the IEEE/CVF Conference on Computer Vision and Pattern Recognition (CVPR).

[33] Shin M., Lee J. (2016). CNN Based Lithography Hotspot Detection. Int. J. Fuzzy Log. Intell. Syst..

[34] Torres J.A. ICCAD-2012 CAD contest in fuzzy pattern matching for physical verification and benchmark suite. Proceedings of the ICCAD.

